# The Neural Basis of Speech Perception through Lipreading and Manual Cues: Evidence from Deaf Native Users of Cued Speech

**DOI:** 10.3389/fpsyg.2017.00426

**Published:** 2017-03-30

**Authors:** Mario Aparicio, Philippe Peigneux, Brigitte Charlier, Danielle Balériaux, Martin Kavec, Jacqueline Leybaert

**Affiliations:** ^1^Laboratory of Cognition, Language and Development, Centre de Recherches Neurosciences et Cognition, Université Libre de Bruxelles,Brussels, Belgium; ^2^Neuropsychology and Functional Neuroimaging Research Unit (UR2NF), Centre de Recherches Cognition et Neurosciences, Université Libre de Bruxelles,Brussels, Belgium; ^3^Department of Radiology, Clinics of Magnetic Resonance, Erasme HospitalBrussels, Belgium

**Keywords:** audiovisual speech perception, lipreading, manual gestures, deafness, Cued Speech, fMRI, MT/V5

## Abstract

We present here the first neuroimaging data for perception of Cued Speech (CS) by deaf adults who are native users of CS. CS is a visual mode of communicating a spoken language through a set of manual cues which accompany lipreading and disambiguate it. With CS, sublexical units of the oral language are conveyed clearly and completely through the visual modality without requiring hearing. The comparison of neural processing of CS in deaf individuals with processing of audiovisual (AV) speech in normally hearing individuals represents a unique opportunity to explore the similarities and differences in neural processing of an oral language delivered in a visuo-manual vs. an AV modality. The study included deaf adult participants who were early CS users and native hearing users of French who process speech audiovisually. Words were presented in an event-related fMRI design. Three conditions were presented to each group of participants. The deaf participants saw CS words (manual + lipread), words presented as manual cues alone, and words presented to be lipread without manual cues. The hearing group saw AV spoken words, audio-alone and lipread-alone. Three findings are highlighted. First, the middle and superior temporal gyrus (excluding Heschl’s gyrus) and left inferior frontal gyrus pars triangularis constituted a common, amodal neural basis for AV and CS perception. Second, integration was inferred in posterior parts of superior temporal sulcus for audio and lipread information in AV speech, but in the occipito-temporal junction, including MT/V5, for the manual cues and lipreading in CS. Third, the perception of manual cues showed a much greater overlap with the regions activated by CS (manual + lipreading) than lipreading alone did. This supports the notion that manual cues play a larger role than lipreading for CS processing. The present study contributes to a better understanding of the role of manual cues as support of visual speech perception in the framework of the multimodal nature of human communication.

## Introduction

There is increasing evidence that sensory-deprived individuals make adjustments to their sensory loss in order to interact effectively within their environment. These adaptations are linked to changes occurring at multiple regions of the brain (Bavelier and Neville, 2002). For people who are deaf from birth or lost their audition early in life, neural plasticity of the regions classically associated with auditory and speech sound processing is related not only to lack of auditory experience but also to the timing and nature of language experience (Cardin et al., 2013). Among the children born deaf, the majority is born to hearing parents, and only 5% have deaf parents. The modality in which language is conveyed can be very different from one deaf child to another: from auditory-oral (listening, talking, and lipreading and facial expressions, known as speechreading), to visual communication strategies like Cued Speech (CS, supporting perception of spoken language with hand shapes that disambiguate lipreading, see below), and/or Sign Language (SL, sign for each language concept, with a grammar of its own). Recently, Olulade et al. (2014) suggested that the nature of language experience (signed vs. oral) has an impact on the development of gray matter volume in the cerebral regions processing language measured in deaf adults, but this point remains to be confirmed.

The timing of language experience can also vary among deaf children. Some of them have daily access to a fully perceivable linguistic input through SL or CS during the first year(s) of life when cerebral plasticity is as its greatest (Kuhl, 2004) while others only have partial access to auditory input (via the cochlear implant) and/or a late access (after the age of 4 years) to visual languages like SL and CS. For instance, deaf children who use SL from early in infancy outperform deaf children who are late learners in tests of SL proficiency and even in tests of English proficiency (Mayberry et al., 2002, 2011). Those children who are exposed to SL only at a later age show long-term language deficits (Emmorey et al., 1995; Lyness et al., 2013). Delayed acquisition of SL as a first language is related to structural changes in the visual cortex (less gray-matter concentration in V1/V2 and V3a/V7 in late signers), and this effect is independent of auditory deprivation (Pénicaud et al., 2013).

Here we tested, for the first time to our knowledge, the language neural processing of congenitally deaf adults who were exposed from early years to spoken language fully perceivable through the visual modality. Since spoken language has evolved to be primarily heard, not seen, critical features like voicing or nasality cannot be perceived by eye only. Among systems dedicated to make spoken language entirely visible to deaf persons, CS is the most widespread one.

**FIGURE 1:**
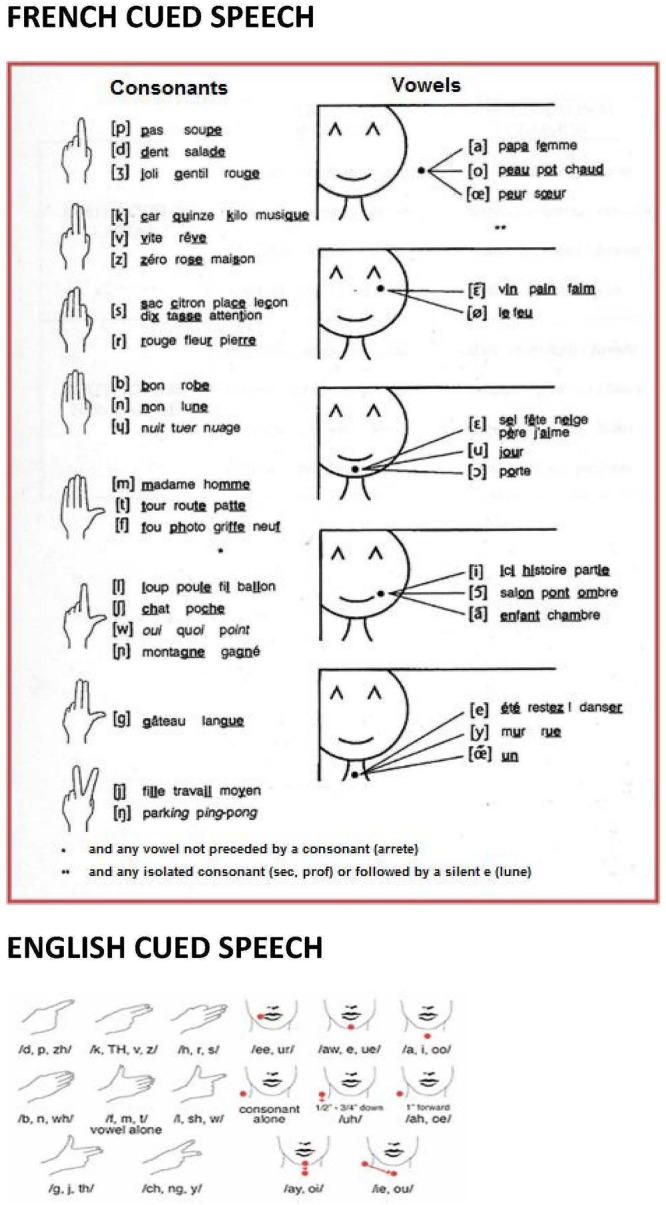
**The French and English Cued Speech codes.** Complete diagram of hand shapes and hand positions of French and English CS. In CS, the speaker holds one hand near the mouth while speaking to complement lipreading with a manual cue. A cue in CS is made of two parameters: hand shape **(left)** and hand position of execution around the mouth **(right)**. For example, syllables as /pa/, /ba/ or /ma/ cannot be distinguished using lipreading because they provide similar visual information. In CS, the syllables /pa/, /ba/ or /ma/ can be easily distinguished by simply using three different hand shapes.

Cued Speech (Cornett, 1967) is a visual communication system used with and among deaf and hard-of-hearing persons. It is a system which delivers consonant-vowel (CV) dyads in the spoken language using a small number of handshapes and locations as a supplement to lipreading (see **Figure 1** for the English and French versions of CS). Handshapes and the location in space where the hand is placed combined with the mouth movements of speech make all the syllables and phonemes of spoken language appear distinct from each other. Consonants that have similar mouth movements (like /p/, /b/ and /m/) are coded with different handshapes (1, 4, and 5, respectively). Consonants that are coded by the same handshape (like /p/, /d/ and /Ʒ/) are easily distinguished by lipreading. Vowels indistinguishable on the lips (for instance French /y/ and /u/) are coded at different hand locations, and the same location is used to code vowels different on the lips.

Each time a speaker pronounces a CV syllable, he/she adds manually information about the word’s phonological structure that is not entirely visible on the lips, by producing a handshape at a particular location in relation to the head and upper body (see **Figure 1**). Take the example of a speaker producing the syllable /pa/. From the lips, the receiver perceives /pa/, /ba/, or /ma/. When the manual cue is added (i.e., handshape n° 1 representing /p, d, j/, produced at the side of the face representing /a, oe, o/ vowels), the uncertainty is reduced, and the syllable /pa/ remains the only possibility. Indeed, /ba/ and /ma/ are eliminated on the basis of the information read from the handshape, and /da/ and /ja/ are incompatible with the information read on the lips. From this example, it clearly appears that CS handshapes and hand locations are not themselves interpretable as syllables or phonemes. The integration of manual and labial information is mandatory to perceive an unambiguous syllable. Deaf users of CS are thus afforded access to the words in a spoken language in which sublexical features are entirely specified solely by visible articulatory gestures, i.e., manual cues and mouth movements.

Cued Speech was created in the 1960s with the aim of allowing deaf children to accurately perceive spoken language and improving their literacy skills (Cornett, 1967; Cornett and Daisey, 2001). However, the overall effect of CS on early spoken language development extends beyond this (Leybaert et al., 2010, 2013, 2015). The empirical evidence collected in English, French, Spanish, Farsi and even Amharic (national language of Ethiopia) shows that congenitally deaf children who were exposed to CS from their earliest months by their parents and other caregivers can reach levels of mastery of spoken (phonology, lexical, morpho-syntactic) language and written language (word reading, reading comprehension, spelling) within range of age-matched hearing peers when tested at school age. Children with late and less intensive exposure (i.e., at the age of 5–6 years, and at school only) do not demonstrate the outstanding phonological and reading abilities of the early CS-users, confirming the existence of a sensitive period for language acquisition via the visual modality (Nicholls and McGill, 1982; Périer et al., 1990; Charlier and Leybaert, 2000; Leybaert, 2000; LaSasso et al., 2003; Torres and Moreno-Torres, 2006; Koo et al., 2008; LaSasso, 2010; Movallali, 2011; Heracleous et al., 2012; Colin et al., 2013; Rees and Bladel, 2013; Shull et al., 2016).

It is this evidence for comparable language abilities reached by congenitally deaf individuals who are early CS-users and NH individuals which raise our interest concerning how the human brain processes linguistic information when conveyed by handshapes and speech-based mouth actions, compared to when conveyed by AV speech. To put this research into perspective, we summarize below critical points about auditory speech processing stream and the way in which lipread signals are integrated with the auditory speech stream. Next the neural basis of visual language perception (SL and speechreading) in deaf participants is discussed. We address the commonalities and differences between SL and CS. Finally, the knowledge about time course of manual and mouth movements articulation in CS is summarized, introducing our three research hypotheses concerning the comparison of neural activation of CS and AV speech.

### Neural Basis of Audiovisual Speech Processing

For hearing people, speech perception is a multimodal phenomenon. It is known since long that vision is of great help for hearing in noise and adverse conditions (Sumby and Pollack, 1954; Erber, 1969). The neuroanatomy and neurophysiology of audiovisual (AV) interactions in the human cortex have been abundantly explored in the last 15 years (see Campbell, 2008 for a review; Calvert et al., 1997, 2000; Calvert and Campbell, 2003). The acoustic speech signal projects posteriorly from Heschl’s convolutions within lateral temporal cortex to further superior temporal regions (secondary auditory cortex). The mid-posterior superior temporal sulcus (pSTS) appears to be a prominent site for AV speech integration (Binder et al., 2000; Callan et al., 2004; Hickok and Poeppel, 2007; Szycik et al., 2007), with yet more posterior regions around the temporo-parietal sulcus being implicated specifically in lip movement perception (see Bernstein and Liebenthal, 2014).

Neuroanatomical studies displayed three types of AV interactions. First, there are direct connections between sensory cortices (Besle et al., 2008). Second, associative areas and particularly the pSTS play a crucial role in AV speech perception (e.g., Beauchamp et al., 2004, 2010). Third, parieto-frontal areas related to speech production are involved through the dorsal route (see Jones and Callan, 2003; Skipper et al., 2005, 2007; Okada and Hickok, 2009). Electrophysiological studies revealed that the influence of visual speech in cortical auditory processing can occur within 100 ms of signal onset, suggesting that lipreading exerts an early effect on auditory signal resolution (Colin et al., 2002; Van Wassenhove et al., 2005; Stekelenburg and Vroomen, 2007; Besle et al., 2008; Arnal et al., 2009; Peelle and Sommers, 2015).

### Neural Activity Related to Lipreading

Since two decades now, the cortical substrates for lipreading in hearing and deaf participants have been widely investigated with neuroimaging techniques. In hearing people, silent lipreading engages activation of pSTS and middle temporal gyrus, and inferior frontal regions (Calvert et al., 1997, 2000; Bernstein et al., 2002; MacSweeney et al., 2000, 2002; Paulesu et al., 2003; Campbell, 2008; Bernstein and Liebenthal, 2014). These regions are similar to those engaged when speech is heard. Activation of left pSTS is correlated with hearing participant’s lipreading skills (Hall et al., 2005).

Some deaf individuals become better lipreaders than normally hearing individuals, i.e., deaf individuals may be better than hearing persons to extract information about spoken language structure from visible lip movements, jaws, and face (Bernstein et al., 2001; Mohammed et al., 2006; Auer and Bernstein, 2007). The variability among deaf individuals is partly explained by their practice and knowledge of oral language. Indeed, those deaf individuals exposed daily to CS from their early years become very proficient lipreaders (Aparicio et al., 2012), likely because precise lipreading is a mandatory component in CS perception.

Variability among deaf lipreaders also appears in neuroanatomical studies. MacSweeney et al. (2000) asked deaf and hearing participants to silently lipread numbers (from 1 to 9) in a scanner. They found that temporal activation was more dispersed on different sites and less intense in the group of deaf participants. They suggested that coherent exposition to AV speech may play an important role for the structuration of temporal cortex for visual speech. In a second study, MacSweeney et al. (2002) found that the cingular cortex is a structure more activated during lipreading in deaf people than in hearing ones. A conjunction analysis of the data of these two studies revealed posterior activation of cingular cortex (BA23/30, related to visuo-spatial functions) in the deaf adults, and a bilateral activation of superior temporal areas (BA22/42) in the hearing adults. The deaf group showed activation of superior temporal gyrus (BA22) on the right side, extending into the tip of Heschl’s gyrus (BA42, part of the secondary auditory cortex), suggesting a predominantly right lateralized network in deaf people.

Capek et al. (2008) realized a further study in which congenitally deaf adults who were native signers and proficient speechreaders and hearing non-signing controls searched for a lipread target (the word “yes”) embedded in lists of silently spoken unrelated words from an open list. Participants pushed a button only when they identified the target word. The control condition was a speaker at rest. The results showed a strong activation of temporal superior gyrus, and in regions located in the Heschl’s gyrus, especially on the left side, in deaf as well as in hearing participants. Inferior frontal gyrus was also activated in both groups, which may reflect the involvement of mirror neurons in lipreading (Campbell et al., 2001; Paulesu et al., 2003; Pekkola et al., 2005). Lipreading ability was assessed outside the scanner, with the Test of Adult Speechreading (TAS, Mohammed et al., 2006). In deaf participants, the neural activations during lipreading were positively correlated with the TAS score. Deaf participants showed greater activation than hearing participants in the left temporal cortex, including the planum temporale and Heschl’s gyrus. Compared to the two previous studies, the Capek et al.’s (2008) one differed on a number of variables (nature of the baseline task, task requirement, stimulus type, and size of the group of deaf participants). These variables can possibly explain the differences in the results obtained (see Capek et al., 2008, p. 1239). The conclusion is particularly relevant to our study: the authors assumed that if the superior temporal cortex is not used to process auditory speech, it may be recruited to process visual speech, to a larger extent than in hearing participants in whom AV speech perception is dominant (see also MacSweeney et al., 2002; Capek et al., 2010 for related data).

Part of the activation induced by lipreading must be related to visual movement detection and to the perception of biological movement, especially in the inferior and posterior regions of the temporal cortex (Zeki et al., 1991). But most of the activation in superior temporal regions is related to lipreading itself. Therefore, the observation of more activation related to lipreading in deaf individuals than in hearing people in the pSTS suggests the following interpretation. The pSTS is an AV integration site in hearing people, but cannot play this role in deaf individuals. The activation of pSTS could be sensitive to the dominant language modality. This multimodal region could have developed sensitivity to visual speech for deaf individuals, and to auditory speech, and secondary to visual speech for hearing ones. Given that our early CS-users participants did not hear during their early years, and have been intensively exposed to lipreading + manual cues in daily communication, one may expect to observe a strong activation of pSTS during CS perception.

### Neural Activity Related to Sign Language

Sign language is the preferred means of communication for most of deaf persons. Deaf persons use visible actions from the hands, the head and the trunk to communicate meanings using phonological, lexical and morpho-grammatical rules. The articulators are visible gestures, and language perception is in the visuo-spatial modality. SLs are adapted to the human’s processing abilities in the visual modality, as are spoken languages in the auditory modality (Christiansen and Chater, 2016). For instance, signs take longer to be articulated, but the mean duration of utterances is similar in SL and in English for ASL-English bilinguals (Bellugi and Fischer, 1972).

Similarities between sign and spoken language processing have been abundantly demonstrated. Lesion-based, neuroimaging, and neurophysiological studies have provided strong evidence for the importance of left perisylvian regions during production and perception-comprehension of signed as well as spoken languages (Emmorey, 2001; Capek et al., 2008; MacSweeney et al., 2008; Corina et al., 2013). The left inferior frontal gyrus is involved in both sign and speech production while the left superior temporal gyrus and sulcus, in addition to the left inferior frontal gyrus, are involved in sign and spoken language perception-comprehension.

Differences related to the visual signal delivered by the articulatory gestures have been revealed between AV seen speech (lipreading) and SL perception (Emmorey, 2001; MacSweeney et al., 2008; Corina et al., 2013; Leonard et al., 2013). The kinematic characteristics of SL and AV speech are very different. There is more movement in the moving image of sign than speech. Different parts of the visual recognition system are sensitive to movements of particular body-parts (mouth specific vs. hand specific regions in inferior temporal cortex, see Pelphrey et al., 2005). The articulators in AV speech and SL thus have different timing, dynamic, and visibility, and their perception elicit different brain activity. SL perception induces greater activation than AV speech in movement processing regions of the posterior temporal gyri, bilaterally, while AV speech perception generates greater activation than SL in auditory processing regions in superior temporal cortices, including the planum temporale (Petitto et al., 2000; MacSweeney et al., 2002).

Of particular relevance for the present study is the fact that some signs require mouth movements in addition to manual movements. Some of these mouth movements allow distinguishing minimal pairs of signs in SL (Sutton-Spence and Woll, 1999; Capek et al., 2008). Activations corresponding to mouth movements, distinct from those related to hand movements have been found during SL perception (Capek et al., 2008). Compared to manual only signs, signs including mouth movement elicit more activation in middle and posterior portions of the superior and middle temporal gyri, and in the inferior and middle frontal gyri, bilaterally. The manual only signs elicited activation in the right occipito-temporal cortex, and the fusiform gyrus. As the moving hand adopting several distinct configurations around the face is a important articulator in CS (see **Figure 1**), one may expect to find more activation in the temporal posterior regions, and perhaps in the temporo-occipital posterior inferior regions in CS than in AV speech.

### Similarities and Differences between CS and SL

Cued Speech, such as cued American English, has similarities and differences with SL. Like SL, CS is conveyed in the visual modality and can be used for social communication, as evidenced by interactions within the family unit, and among “cuers” in social events. Similarly to SL, the use of voice is not needed in order to communicate in CS: “cuers” can achieve 100% speech perception when manual cues and mouth movements are presented, without sound (Nicholls and McGill, 1982). Finally, for SL as for CS, presentation of a single phonological parameter of the lexeme cannot, on its own, generate a lexical item (similar handshapes and hand locations occur with multiple lexemes in CS, as similar handshapes do in SL). Even more relevant to the present study is the fact that some signs requiring mouth actions in SL cannot be interpreted unambiguously at the lexical level when presented in isolation (see Capek et al., 2008). This is also the case in CS where the manual cues cannot, on their own, generate access to a lexical item. Accurate perception of CS thus necessarily involves the integration of manual and labial information in order to achieve a specific lexical representation. That is, for CS as well as for SL, individual sublexical features of articulation must be integrated to allow access to a specific lexical representation.

There are also differences between CS and SL. First, CS is not a language, but rather a visual mode of communication of spoken language. CS is isomorphic to speech: it is a visual representation of the syllables and phonemes of spoken language while SLs have their own phonology based on manual articulatory parameters of hand location, handshapes, movement, and palm orientation (Stokoe, 1960). Second, CS handshapes are produced at a much more rapid rate than SL. The production of handshapes at different locations around the face follows the rate of spoken speech, meaning that the CS receiver must decode a rapid succession of changing handshapes and hand placements, in a space located from the speaker’s eyes to the throat (Attina et al., 2004, 2006). In order to formulate more precise predictions about activations related to CS perception, it may be useful to know what has been revealed so far about the time course of mouth and hand movements in CS. This is the topic of the next section.

### What Do We Know about Perception and Production of CS?

Attina et al. (2004) were the first to examine the precise temporal organization of CS production of syllables, words and sentences. Natural production of CS is characterized by a temporal anticipation of manual gesture over mouth opening: the hand movement begins up to 240 ms before the acoustic onset of a CV syllable, and the target position corresponding to the vowel is reached during the mouth production of the consonant, well before the vowel lip target. At the receptive level, deaf CS-users anticipate the linguistic target on the basis of “reading” the manual gesture: perception of the hand gives the first input for the selection of the possible phonemes pronounced, and the lips follow with the solution. Deaf people seem to extract first phonological information when a manual cue is produced, reducing the potential number of words compatible with the lipread signal. Attina et al. (2004) data suggest that manual cues, as opposed to lipread information, can be the primary source of phonological information for deaf early CS users. Lipread information would then disambiguate the information provided by the manual cues (Attina et al., 2004, 2006; Troille et al., 2007; Troille, 2009).

### Predictions Concerning the Neural Activation for AV Speech versus CS Perception?

In this study, we compare the neural activations created by processing of spoken language produced and perceived through two different modalities: AV and CS. We control for language experience by testing only native users: NH participants with the AV material, and congenitally deaf participants with the CS material. We address three research questions. First, what are the similarities and differences between the processing of CS by early CS-users and the processing of AV speech by NH participants? This comparison is designed to explore common regions of activation for spoken language, independent of modality. Our second research question concerns the neural basis for integration of manual cues and lipread information. In CS, integration is mandatory and concerns two types of dynamic visible information, i.e., the movements of labial and manual articulators. In AV speech, integration concerns two modalities that are congruent in terms of their articulatory origins: the heard and seen results of movements of the oral articulators. We expected to find the pSTS as site of integration for AV speech, and we wanted to document brain regions critical for CS integration. Our third research question concerned the relative activation created by manual cues only and lipreading cues only compared to the activation created by the combined movements of lips and hands in CS. Some authors have suggested that the manual component of CS delivers more useful information than the lipread component to get access to the lexicon (Alegria et al., 1999; Alegria and Lechat, 2005; Attina et al., 2006; Troille, 2009; Bayard et al., 2014, 2015). We hypothesize that the cortical activations may reveal greater activation for unisensory manual than labial movements, but also indicate specific locus/loci for integration of manual and labial information, different from those reported for AV integration.

## Materials and Methods

### Ethics Statement

All participants gave written informed consent to participate in this study, which was approved by the Ethics Committee of the ULB Erasme Hospital, and conducted in accordance with the Declaration of Helsinki (BMJ 1991; 302: 1194).

### Participants

Two groups of participants were recruited. The *CS group* consisted of 14 participants (3 males, 11 females), with a mean age of 25.0 years (age range = 18–33 years). All participants but one were congenitally profoundly deaf, with a binaural hearing loss > 90 dB (computed on 250, 500, 1000, and 2000 Hz) in their better ear. The remaining participant had a severe hearing loss (i.e., between 71 and 90 dB at the better ear). All deaf participants were equipped with hearing aids since they were between 6 months and 2 years of age, and none had a cochlear implant.

The *NH group* consisted of 15 normally hearing French-speaking participants (six males, nine females), with no knowledge of CS. Their mean age was 25 years 2 months (age range = 20–37 years).

Since native language involves a different brain network than second languages learned later in life (Dehaene et al., 1997), only participants who were native French speakers were selected. A participant was considered a native language user if he/she had received consistent, age-appropriate speech stimulation from fluent users of French before the age of 3 years (Locke, 1997). Currently, this criterion for native CS user can only be fulfilled within the deaf community, since nearly all NH people with an experience in French CS learned it later in their life. Consequently, only neural activity from deaf CS participants who were exposed at an *early (i.e., prelingual) age* is an appropriate comparison for the patterns of neural activity observed in native French speaking hearing participants.

The deaf participants of the CS group in our study self-reported French as their native language in a questionnaire completed prior to enrolling in the study. They had been exposed to French CS, at home from their parents before the age of 3 years, and at school via teachers and or via transliterators from spoken French to French CS. Participants also reported that French CS was the language most commonly used during their childhood/adolescence, although most of them also learned SL informally during this period in contacts with deaf peers. The CS users reported that they still use CS often today, in daily communication with their family or other deaf persons. They also used oral French to communicate with NH individuals. The NH transliterator gave qualitative feedback about deaf participants’ CS comprehension: all of them could easily understand normal cued French conversation and were good at lipreading. All CS participants had finished secondary school (high school), and 50% (*n* = 7) had either completed a post-graduate program or were in one at the time of the testing.

All deaf and hearing participants were right handed, with no known neurological or behavioral disorder.

### Experimental fMRI Design (Procedure)

In an event-related paradigm, a baseline condition and three experimental conditions were presented randomly intermixed to each group of participants. For deaf participants, the experimental conditions consisted of stimuli presented in CS (lipreading + manual cues), in lipreading alone, or in handshapes alone. For NH participants, the experimental conditions consisted of stimuli presented in AV, in auditory speech alone, or in lipreading alone. For both groups, the baseline condition consisted of a motionless face.

#### Experimental Task and Conditions

In the experimental conditions, all participants watched videos of a hearing female French speaker (who was a professional CS transliterator) saying a randomized list of 45 bisyllabic frequent words (New et al., 2001) (see Annex for the full list of words used in this experiment) under six conditions (three for the NH group and three for the CS group; see below). All videos were recorded indoor (in a room). The female speaker was so positioned that her back was in contact with a white wall. She looked straight to the camera. Her full face and torso were shown in all videos in order to present a naturalistic display of CS.

In the *NH group*, participants performed the detection task under three different experimental conditions: (1) the speech AV condition, in which the speaker pronounced the word while the same word was presented orally through MR-compatible earphones, (2) the auditory alone (A) condition, in which the word was presented aurally but the speaker’s face remained motionless, and (3) the lipreading alone (L) condition, in which the speaker pronounced the word but no auditory information was provided. In the *CS group*, participants performed also the detection task under three different experimental conditions: (1) the Cued Speech labial + manual (CSLM) condition, in which both lip movements and manual cues were visible, (2) the CS manual (CSM) condition, in which only hand movements were provided, but the speaker’s face remained still (no lips movements), and (3) the CS labial (CSL) condition, equivalent to lipreading, in which the speaker pronounced the word, but no manual movements were provided.

For each condition, one video corresponded to one word. Altogether, there were 270 videos (i.e., 45 for each condition). Importantly, there was no manipulation of videos from one condition to another. For example, for the CSM condition, we did not create CS manual videos removing the lips information from the CS labial + manual videos. Instead, we recorded 45 videos in which the speaker had to produce the 45 words using only hand movements. Seemingly, in the CSL condition (lipreading condition), we recorded 45 videos in which the speaker pronounced the word without hand movements. The dynamic of both, hand movements produced in CSM videos and lip movements produced in CSL videos, have a great likeness when compared to the CSLM condition.

The same words were used in all conditions but in different orders between conditions. In each of the three conditions, the target word “papa” (i.e., daddy) was included in the list of stimuli. In order to ensure focused attention to the stimuli, participants were asked to press a button when they detected “papa” (i.e., daddy).

#### Control Task – Baseline Condition

In order to control for attention, motor response parameters, and perception of a face and a body, a second task was designed. In this control task, all participants saw a still picture of the speaker’s full face and torso on screen, as in the experimental task (45 trials). They had to press a button when a small red circle was superimposed on the speaker’s chin. The group conditions are summarized in **Table 1**.

**Table 1:** Contrasts of the experimental conditions and the control condition.

	CS group	NH group
Experimental conditions	(1) Cued Speech oral + manual (CSLM)	(1) Speech audiovisual (speech AV)
	(2) Cued Speech manual only (CSM)	(2) Speech auditory (speech A)
	(3) Cued Speech oral only (lipreading) (CSL)	(3) Speech visual (lipreading) (speech V)
Control condition	Still	Still

#### Procedure

Before the presentation of each video, a white cross was displayed in the middle of the screen, indicating that the stimulus material (i.e., the video) would appear randomly within 1900–2800 ms. Then, the video containing the stimuli was displayed. The length of each video was also randomly assigned within 1900 and 2800 ms but, for all six conditions, they had the same mean of 2370 ms and the same standard deviation of 220 ms. In order to do this, we recorded each video with a length of 3 s, but leaving always a silent space at the end of at least 1200 ms in which the speaker’s face and torso remained present but still. This allowed us to adjust the required length for each video (by cutting the necessary time needed for each video). After the presentation of each video stimulus, a question mark appeared on the screen for 2000 ms, reminding the participant to press a button on the MR-compatible keypad (fORP; Current Designs) only if the participant had perceived the target word (i.e., “papa”), in one of the experimental conditions, or the target circle in the still control task. The target word or red circle appeared eight times within each condition.

Presentation of the stimuli was randomized across the three experimental and the control conditions. Presentation of the stimuli and recording of the participant’s responses were realized using Cogent Graphics (running on Matlab^TM^ 6.1) developed by John Romaya at the LON at the Wellcome Department of Imaging Neuroscience. All participants practiced the tasks outside of the scanner beforehand to minimize task-learning effects. fMRI data acquisition had an approximate duration of 30 min.

### fMRI Data Acquisition

Data were acquired on a Philips Achieva 3-T (Philips Medical Systems, Best, the Netherlands) scanner using a T2^∗^ sensitive gradient echo (EPI) sequence (TR = 2130 ms, TE = , 40 ms, FA 90°, SENSE acceleration factor 2.5, matrix size 64 × 64 × 32; voxel size: 3.06 mm × 3.06 mm × 3 mm). Thirty-two contiguous 3-mm thick transverse slices were acquired, covering the whole brain. An approximate number of 840 EPI volumes per participant was acquired across the four conditions. Additionally, an anatomical image was obtained using a T1-weigthed sagittal 3D MP-RAGE sequence (TR 1960 ms, TE 4.60 ms, TI 1040 ms, flip angle 8°, FOV 250 mm × 250 mm, matrix size 320 × 320 × 160, interpolated voxel size: 0.78 × 0.78 × 1.0 mm). The MR scanner was equipped with the Quasar imaging gradients and an eight channel SENSE head coil.

### fMRI Data Analysis

Functional MRI data were pre-processed and analyzed using Statistical Parametric Mapping (SPM8) software (Wellcome Department of Cognitive Neurology, London, UK) implemented in MATLAB 7.8 (Mathworks Inc., Sherbom, MA, USA). The first five functional EPI volumes were discarded to avoid magnetic saturation effects. The remaining EPI images were realigned (Woods et al., 1992), spatially normalized into standard stereotactic MNI space (Woods et al., 1999), and smoothed spatially at 8 mm full-width half-maximum (FWHM) (Worsley et al., 1997).

Data were analyzed using a mixed-random effects (RFX) model aimed at showing a stereotypical effect in the population from which the subjects were drawn (Penny and Holmes, 2003). For each subject, a first-level, intra-individual analysis aimed at modeling data to partition observed neurophysiological responses into components of interest, confounds, and errors, using a general linear model (Friston, 2003). The regressors of interest were built using stick functions corresponding to the four conditions (45 stimuli each). These regressors were secondarily convolved with a canonical hemodynamic response function. Movement parameters derived from realignment of the functional volumes (translations in x, y, and z directions and rotations around x, y, and z axes) were included as covariates of no interest in the design matrix. High-pass filtering was implemented in the matrix design using a cut-off period of 128 s to remove low drift frequencies from the time series. Serial correlations were estimated with a restricted maximum likelihood (ReML) algorithm using an intrinsic autoregressive model during parameter estimation. Effects of interests were then tested by linear contrasts (e.g., CSLM – still, Speech AV – still, CSLM vs. Speech AV, etc.), generating statistical parametric maps [SPM(T)].

Individual summary statistic images were further spatially smoothed (6 mm FWHM Gaussian kernel) and entered in a second-level analysis in which participants were treated as a RFX. At this second level, one-sample *t*-tests were used to assess the contrasts between two conditions in the CS and NH groups separately. Two-sample *t*-tests were used for a direct comparison of the same contrasts between CS and NH participants. Additionally, conjunction null analyses (Price and Friston, 1997; Friston et al., 2005) were used to identify the brain areas commonly activated between conditions or between CS and NH groups in contrasts of interest. This method tests whether individual effects are jointly significant, under the null hypothesis that all-but-one of the effects are significant, hence an “AND” over several individual hypotheses. ReML estimates of variance components were used to allow possible departure from the sphericity assumptions in RFX conjunction analyses (Friston et al., 2002).

In order to explore the role of MT/V5 region to the experimental conditions (see below, p 21), psychophysiological interaction (PPI) analyses (Friston et al., 1997; Gitelman et al., 2003) were computed to test the hypothesis that experimental conditions modulate functional connectivity between neural activity in left or right MT/V5 areas and other brain regions involved in CS and AV processing. First, the time course of activity within MT/V5 area was extracted separately at left and right MT/V5 coordinates for each individual. To do so, the CSLM (respectively speech AV) vs. still contrast effect (corresponding to the summary statistic images entered in the RFX analysis) was computed at the individual level, and the local maximum of activation determined in a volume within the probabilistic map of MT/V5, as identified in a previous cytoarchitectonic analysis (Malikovic et al., 2007). This peak value was selected, unless it was identified outside of the brain structure of interest upon visual inspection of the individual normalized anatomical T1 image and verification of localization in SPM toolbox Anatomy atlas (Eickhoff et al., 2005), in which case the maximum value that fitted the anatomical location was selected. Second, a new linear model was generated for each individual level, using three regressors. One regressor represented the task condition (CSLM [respectively speech AV] vs. still). The second regressor was the average activity in a sphere (radius 4 mm) centered on the coordinate of the participant-specific peak value. The third regressor represented the interaction of interest between the first (psychological) and the second (physiological) regressor. To build this regressor, the underlying neuronal activity was first estimated by a parametric empirical Bayes formulation, combined with the psychological factor (i.e., task condition) and subsequently convolved with the hemodynamic response function (Gitelman et al., 2003). The design matrix also included the movement parameters caused by subject movements. A significant PPI indicated a change in the regression coefficients between any reported brain area and the reference region related to the task condition. Individual summary statistic images obtained at the first level (fixed effects) analysis were then spatially smoothed (6 mm FWHM Gaussian kernel) and entered into a second-level (RFXs) analysis using one sample *t*-tests to test for condition-specific effects within CS or NH group separately.

Additionally, we performed an integration analysis, in which we searched for brain areas more strongly activated in a bi-articulatory condition (e.g., CSLM) than in the mono-articulatory conditions (e.g., CSM and CSL), during which only one of the two components was manipulated. This criterion of super-additivity (Beauchamp, 2005) was implemented as a t-contrast between the bi-articulatory condition and the sum of the two mono-articulatory conditions [e.g., CSLM > (CSM + CSL)]. For the CS group, the null hypothesis was thus a weighted contrast [2 -1 -1] such as [2^∗^(mean activity during CSLM)] vs. [mean activity during CSM + mean activity during CSL]. For the NH group, the null hypothesis was [2^∗^(mean activity during speech AV)] vs. [mean activity during speech auditory + mean activity during speech visual]. This comparison was masked inclusively by contrasts computed in the mono-articulatory conditions (e.g., CSM > still and CSL > still). This procedure indicated regions mainly devoted to integration while controlling for movement-related activity from manual cues and lipreading. This latter aspect is important since lip movements may have influenced activation results in simple subtraction analyses (e.g., CSLM-still).

In all of the analyses presented above, the resulting set of voxel values for each contrast constituted a map of the t-statistic [SPM(T)], at *p* < 0.001 threshold (uncorrected for multiple comparisons). Statistical inferences were then obtained after corrections at the voxel level using Gaussian random field theory (Worsley et al., 1996), *p*^corr^< 0.05 corrected for multiple comparisons in the whole brain volume, unless otherwise specified.

Anatomical localization of local maxima and clusters was assessed with reference to the MNI space, using standard anatomical atlases (Collins et al., 2002). MT/V5 is a functional area that was located using a probabilistic map in the Anatomy SPM Toolbox (Eickhoff et al., 2005; Malikovic et al., 2007).

## Results

### Behavioral Data

The target detection of the word “papa” was accurate in both groups for all experimental conditions. In the CS group the mean performance was 100%, 99.1% (*SD* = 3.34) and 96.4% (*SD* = 5.8) in the CSLM, CSM and CSL conditions respectively. In the NH group, the mean performance was 100%, 95% (*SD* = 11.3) and 91% (*SD* = 19.5) in the speech AV, speech auditory and speech visual conditions respectively. The mean performance for the detection of the small circle in the control still task was 91% (*SD* = 12.9) in the CS group and 95% (*SD* = 11.7) in the NH group. The mean global performance was 98.5% in the deaf-CS group and 95.3% in the NH group. There was no significant difference between groups (*t* = 1.07; n.s.).

### fMRI Data

#### Brain Activation during CS Processing: CSLM-Still (Deaf)

In CS participants, CSLM perception elicited higher blood-oxygen level dependent (BOLD) responses than the control (still) condition bilaterally in the occipito-temporal junction including the MT/V5 area, and in the middle and superior temporal lobe with a more extended activation in the left than in the right hemisphere. The activation in the superior temporal lobe did not include the primary auditory cortex (Heschl’s gyrus). Other areas activated within this contrast (CSLM-still) were the left inferior parietal lobe, the premotor area, and the inferior frontal gyrus, *pars triangularis* (BA 45).

#### Brain Activation during Speech AV Processing: Speech AV-Still (Hearing)

**FIGURE 2:**
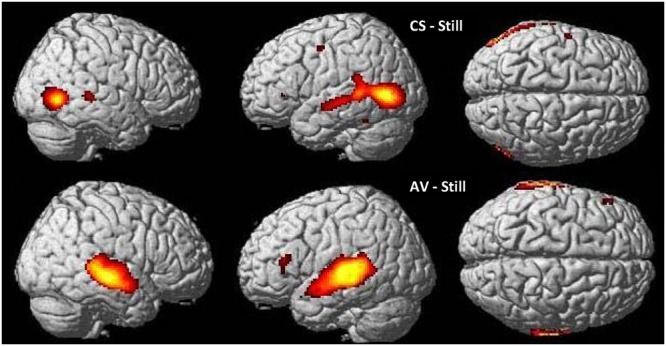
**Word perception in CSLM and speech AV.** Higher activations during CSLM and speech AV perception of words as compared to the still control condition. All activations are displayed at *p* < 0.05 whole brain corrected, superimposed on the SPM MRI template. CSLM, Cued Speech (oral + manual) in deaf early users; AV, audiovisual speech in hearing speakers.

In NH participants, speech AV perception elicited greater BOLD responses than the control condition in bilateral superior and middle temporal gyri, including primary auditory cortex (Heschl’s gyrus), as well as in bilateral inferior parietal lobe and left inferior frontal gyrus, *pars triangularis* (**Figure 2** and **Table 2**).

**Table 2:** Brain activation during CSLM and speech AV word processing, related to **Figure 2**.

Cerebral region – cluster	Coordinate of the peak significant activation (*p* < 0.05, FWE corrected) and cluster extent							
	Speech AV-still (NH group)				CSLM – still (CS group)			
	voxels	*x*	*y*	*z*	voxels	*x*	*y*	*z*
L Superior temporal gyrus *not including primary auditory cortex* L Middle temporal gyrus L Occipito-temporal junction (MT/V5) L Middle occipital gyrus L Inferior occipital gyrus L Inferior temporal gyrus L Inferior parietal lobe					2294	–54	–74	4
L Middle temporal gyrus L Superior temporal gyrus *including primary auditory cortex* L inferior Parietal lobe	3216	–64	–20	2				
R Occipito-temporal junction (MT/V5) R Inferior occipital gyrus R Inferior temporal gyrus R Middle occipital gyrus R Middle temporal gyrus					800	52	–68	2
R Middle temporal gyrus R Superior temporal gyrus *Including primary auditory cortex* R Inferior parietal lobe	2448	62	–22	6				
L Inferior Frontal gyrus *pars triangularis* (BA 45)	86	–50	28	14	4	–50	32	2
R Middle temporal gyrus R Superior temporal gyrus					135	52	–34	2
L Precentral gyrus (BA 6)					23	–56	–4	48
L Inferior temporal gyrus					12	–50	–48	–24

*Brain regions more activated in the word perception task (speech AV or CSLM) than in the still control task. Only peak activations for distinct anatomical structures are reported within each cluster. L, Left; R, Right; BA, Brodmann Area; x, y, z are standard stereotaxic coordinates in the MNI (Montreal Neurological Institute) system. All activations reported at p < 0.05 whole brain corrected. CSLM, Cued Speech (oral + manual). AV, audiovisualin hearing speakers.*

#### Overlapping Brain Activations: A Conjunction Analysis of CSLM and Speech AV Processing

**FIGURE 3:**
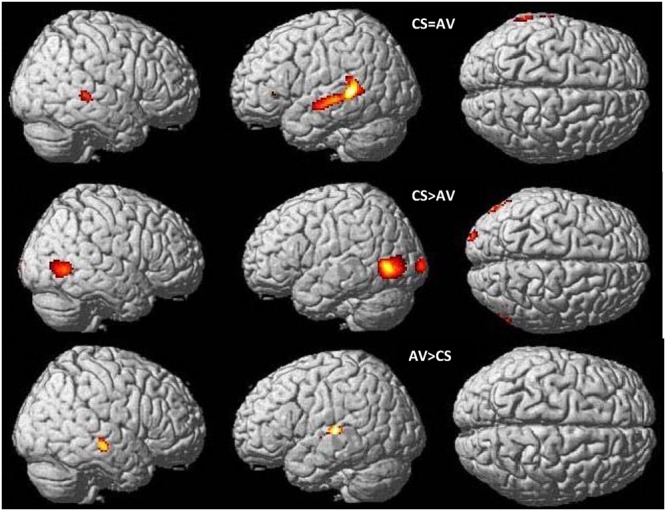
**Common and distinct patterns in CSLM and speech AV during word perception.** Conjunction analysis between speech AV [speech AV – still] and CSLM [CSLM – still] perception of words **(top)**. Higher activations during CSLM perception of words [CSLM – still] compared to speech AV perception of words [speech AV – still] **(middle)** and during speech AV perception of words [speech AV – still] compared to CSLM perception of words [CSLM – still] **(bottom).** Conjunction analysis and CSLM > speech AV activations are displayed at *p* < 0.05 whole brain corrected while speech AV > CSLM is displayed at *p* < 0.001 uncorrected. All are superimposed on the SPM MRI template. CSLM, Cued Speech (oral + manual) in deaf early users; CSM, Cued Speech (manual only); AV, audiovisual speech in hearing speakers.

A conjunction analysis revealed a common activation pattern for AV speech (NH group) and CSLM (CS group) bilaterally in the middle and superior temporal gyrus (excluding Heschl’s gyrus) and in the left inferior frontal gyrus *pars triangularis* (**Figure 3** and **Table 3**), indicating that, as in speech AV perception, CSLM perception is associated with neural activation in the secondary auditory cortices despite a complete absence of auditory input.

**Table 3:** Commonalities (conjunction analysis) and differences (two sample *t*-test) between CSLM (CS group) and speech audiovisual (NH group) processing, related to **Figure 3**.

Cerebral region – cluster	Coordinate of the peak significant activation (*p* < 0.05, FWE corrected) and cluster extent								
	Speech AV = CSOM^1^				CSLM > speech AV ^2^				Speech AV > CSLM^3^			
	Voxels	*x*	*y*	*z*	Voxels	*x*	*y*	*z*	Voxels	*x*	*y*	*z*
L Middle temporal gyrus L Superior temporal gyrus Left Inferior parietal lobe	780	–64	–22	–4								
L Occipito-temporal junction (MT/V5) L Middle occipital gyrus L Inferior occipital gyrus L Inferior temporal gyrus					732	–56	–76	0				
R Middle temporal gyrus R Superior temporal gyrus	135	52	–34	2								
R Occipito-temporal junction (MT/V5) R Inferior occipital gyrus R Inferior temporal gyrus					374	54	–62	0				
L Middle occipital gyrus					167	–24	–102	2				
R Middle temporal gyrus									88	64	–22	–10
L Superior temporal gyrus *Including primary auditory cortex*									107	–60	–24	8
L Inferior frontal gyrus *pars triangularis*	4	–50	32	2								

*^1^Regions commonly activated in both conditions: [speech AV – still control condition] and [CSLM – still control condition] perception of words. ^2^Brain regions more activated during CSLM perception of words [CSLM vs. still control condition] compared to speech AV perception of words [speech AV – still control condition]. ^3^Brain regions more activated during speech AV perception of words [speech AV vs. still control condition] compared to CSLM perception of words [CSLM – still control condition]. L, Left; R, Right; *x, y, z* are standard stereotaxic coordinates in the MNI (Montreal Neurological Institute) system. Activations of speech AV = CSLM and speech AV > CSLM are reported at p < 0.05 whole brain corrected. Activations of CSLM > speech AV are uncorrected for multiple comparisons; p < 0.001. Speech AV = audiovisual speech in hearing speakers; CSLM, Cued Speech (oral + manual); CSM, Cued Speech (manual only); CSL, Cued Speech (oral only).*

#### Differences in Brain Activation between CSLM and Speech AV Word Processing: A Two Sample *t*-test Analysis

In comparing the speech AV and CSLM networks, we found greater BOLD responses in the CSLM (CS group) than in the speech AV (NH group) condition bilaterally in the occipito-temporal junction (MT/V5) and neighboring structures, with a greater extension in volume in the left hemisphere. Greater activations for speech AV than CSLM was found in the right middle temporal gyrus and the left superior temporal gyrus including Heschl’s gyrus (**Figure 3** and **Table 3**).

#### Is MT/V5 Involved in Speech Processing? Psychophysiological Interaction Analysis (PPI) in MT/V5 for CSLM and Speech AV Word Processing

**FIGURE 4:**
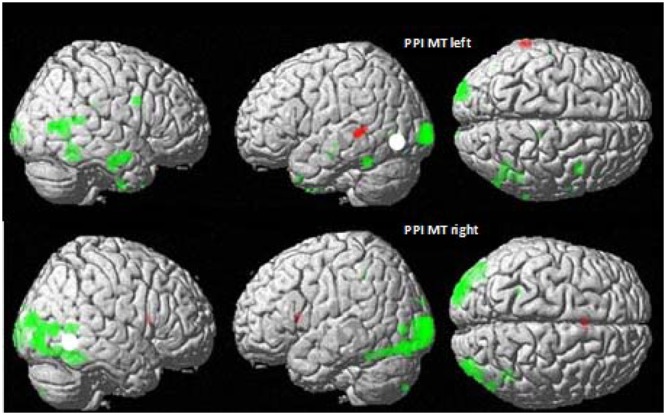
**MT/V5 Psychophysiological interactions in CSLM and speech AV.** Psychophysiological interactions (PPI) from the left **(top)** and right MT/V5 **(bottom)** for deaf CS group (in green) and NH group (in red). *p* < 0.001 uncorrected for multiple comparisons.

The results shown above indicate that besides large commonalities, the neural basis of speech perception in CSLM in CS participants is shifted toward posterior regions of the brain as compared to speech AV in NH participants, with activation peaks in the occipito-temporal junction and MT/V5. Admittedly, activation in MT/V5 is associated with visual motion processing (Zeki et al., 1991; Tootell et al., 1995; Zacks et al., 2001), which was present in the CSLM but not in the control still condition. To test further whether activation in MT/V5 was associated specifically with processing of CSLM, a PPI analysis was performed with MT/V5 (left and right) as source area and CSLM vs. still condition as modulatory parameters. Results revealed increased connectivity during CSLM perception as compared to the still condition between the *left MT/V5* and temporal lobes in both hemispheres, as well as with the posterior inferior temporal and fusiform gyrus bilaterally (**Figure 4** and **Table 4**).

**Table 4:** Psychophysiological Interactions from left and right MT/V5 in NH and CS groups, related to **Figure 4**.

Cerebral region – cluster	Coordinate of the peak significant activation (*p* < 0.05, FWE corrected) and cluster extent							
	NH group				Deaf CS group			
	Voxels	*x*	*y*	*z*	Voxels	*x*	*y*	*z*
**Left MT/V5**								
L Middle occipital gyrus (V3; BA 17/18)					348	–22	–102	1
R Occipito-temporal junction (MT/V5) R Middle temporal gyrus					256	40	–62	9
R Middle temporal gyrus R Inferior temporal gyrus					158	54	–10	–17
R Inferior temporal gyrus R Fusiform gyrus					98	42	–56	–5
L Inferior temporal gyrus L Fusiform gyrus					59	–46	–50	–17
R Precentral gyrus					46	44	4	35
R Fusiform gyrus					23	38	–12	–37
L Middle temporal gyrus					4	–60	–22	–5
L Middle temporal gyrus					4	–50	–18	–11
L Temporal pole					3	–48	8	–31
L Middle temporal gyrus	48	–68	–44	7				
**Right MT/V5**								
L Middle occipital gyrus (V3; V4) L Fusiform gyrus					1032	–28	–102	1
R Inferior occipital gyrus R Middle temporal gyrus R Fusiform gyrus					598	38	–62	–7
R Middle occipital gyrus					144	36	–94	17
R Inferior parietal lobe					73	42	–54	6
L Middle occipital gyrus (MT/V5)					24	–48	–78	2
L cerebellum					20	–16	–82	–48
R Fusiform gyrus					10	28	–42	–20
L Superior parietal lobe					6	–26	–44	48

*L, Left; R, Right; BA, Brodmann area; *x, y, z* are standard stereotaxic coordinates in the MNI (Montreal Neurological Institute) system. All activations reported at *p* < 0.05 whole brain corrected. AV, audio-visual; CS, Cued Speech.*

Increased connectivity between left MT/V5 and temporal regions typically activated for speech processing suggests that left MT/V5 interacts with regions playing a role during CSLM perception. *Right MT/V5* was associated more strongly with CSLM in more posterior brain regions, including the middle occipital and fusiform gyri bilaterally, the left MT/V5 and superior parietal lobe, the right inferior occipital and posterior middle temporal gyri, and the right inferior parietal lobe.

Similar PPI analyses conducted in the NH group revealed a higher connectivity during speech AV than still condition between the left MT/V5 and a small left middle temporal region (**Figure 4** and **Table 4**).

#### Where Does Integration of Manual Cues and Lipreading in CSLM Take Place? Super-Additivity Analysis of Speech AV and CSLM Word Processing

**FIGURE 5:**
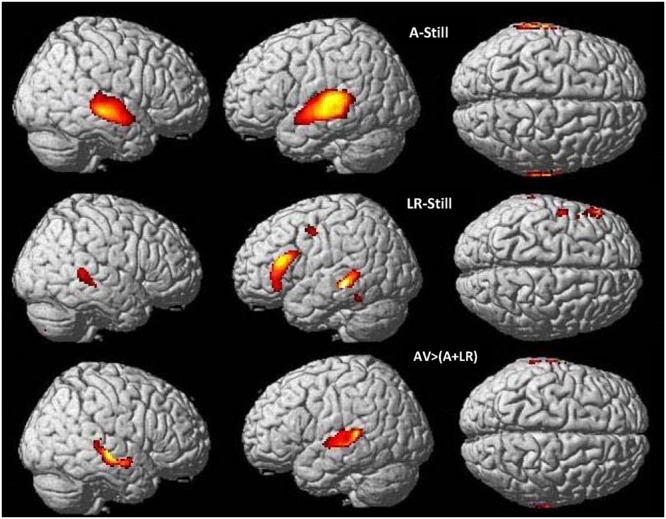
**Neural basis of lipreading and auditory integration in AV hearing speakers.** Higher activations during speech auditory perception of words compared to the still control condition **(top)** and speech visual perception of words (lipreading) compared to the still control condition **(middle)** in AV hearing French speakers. Integration analysis of speech auditory and speech visual (lipreading) in speech AV: higher activations during speech AV [speech AV – still] compared to the sum of [speech A – still] and [speech visual – still] **(bottom)**, masked by contrasts of the uni-articulatory conditions (speech A > still and speech visual > still) in speech AV hearing French speakers. AV, audiovisual speech in hearing speakers; A, auditory; *p* < 0.05 whole brain corrected.

We performed analyses to identify the brain regions responding more strongly to bi-articulation (CSLM in the CS group; speech AV in the NH group) than to the sum of the mono-articulation stimulus presentation (e.g., CSM and CSL separately). The integration analysis in the NH group [speech AV – (speech auditory + speech visual)] revealed greater activity in the bilateral posterior superior and middle temporal gyri (**Figure 5** and **Table 5**). This result is in agreement with findings from previous studies of spoken language that have shown that integration between lipreading and auditory speech takes place in the pSTS (Callan et al., 2004; Szycik et al., 2007; Beauchamp et al., 2010, but see Hocking and Price, 2008). It is hypothesized that the particular sensitivity of this area for multimodal speech integration arises from a correlation between the dynamic aspects of *seen* lipreading and *heard* auditory speech (Calvert and Campbell, 2003).

**Table 5:** Brain activation during processing of auditory condition, lipreading condition and integration in NH group, related to **Figure 5**.

Cerebral region – cluster	Coordinate of the peak significant activation (*p* < 0.05, FWE corrected) and cluster extent								
	Speech A-still^1^				Speech visual (LR)-still^2^				Speech AV integration^3^			
	Voxels	*x*	*y*	*z*	Voxels	*x*	*y*	*z*	Voxels	*x*	*y*	*z*
L Middle temporal gyrus L Superior temporal gyrus *Including primary auditory cortex* L Inferior parietal lobe	2504	–62	–26	6								
L Superior temporal gyrus									559	–60	–28	4
L Middle temporal gyrus					357	–58	–36	–2	444	–62	–20	6
L Inferior temporal gyrus					25	–50	–46	–18				
R Middle temporal gyrus R Superior temporal gyrus *Including primary auditory cortex* R Inferior parietal lobe	1344	64	–14	–8								
R Middle temporal gyrus					222	48	–40	4				
L Inferior frontal gyrus *pars opercularis* (BA 44) *pars triangularis*					702	–48	28	14				
L Precentral gyrus (BA 6)					49	–54	–4	46				
R Superior temporal gyrus	8	48	–34	10					10	40	–34	0

*^1^Brain regions more activated during speech auditory (only) perception of words compared to still control condition. ^2^Brain regions more activated during speech visual (lipreading) perception of words compared to still control condition in AV hearing speakers. ^3^Brain regions activated during AV integration in hearing speakers: [speech AV > (speech A + speech visual)] Ω (speech A > still) Ω (speech visual > still). Activations of speech A-still and speech visual-still are reported at p < 0.05 whole brain corrected. Activations of speech AV integration are uncorrected for multiple comparisons; p < 0.001. AV, audio-visual; A, auditory; LR, lipreading. L, Left; R, Right; BA, Brodmann area; x, y, z are standard stereotaxic coordinates in the MNI (Montreal Neurological Institute) system.*

**FIGURE 6:**
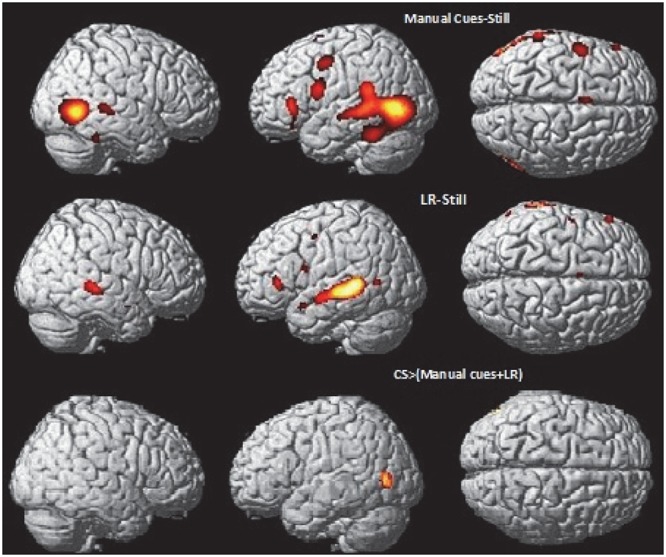
**Neural basis of lipreading and manual cue integration in deaf CS group.** Higher activations during manual cue perception of words compared to the still control condition **(top)** and lipreading perception of words compared to the still control condition **(middle)** in deaf early users of CS. Integration analysis of CSM and CSL (lipreading) in CSLM: higher activations during CSLM [CSLM – still] compared to the sum of [CSM – still] and [CSL – still] **(bottom)** masked by contrasts of the uni-articulatory conditions (e.g., CSM > still and CSL > still) in deaf early users of CS. CSLM, Cued Speech (oral + manual); CSM, Cued Speech (manual only); CSL, Cued Speech (oral only); *p* < 0.05 whole brain corrected.

In the CS group, the integration analysis [i.e., CSLM ≥ (CSM + CSL)] revealed a supplementary activation in the left occipito-temporal junction only, around the MT/V5 area (**Figure 6** and **Table 6**). This is the same location as the activation peak in the CSLM ≥ still condition (see **Figure 2** and **Table 2**). This finding further suggests that the left occipito-temporal junction, including MT/V5, supports the integration of lipread and manual speech features, above a mere visual processing of motion input.

**Table 6:** Brain activation during processing of CSM, CSL (lipreading) and integration of CSLM in deaf-CS group, related to **Figure 6**.

Cerebral region – cluster	Coordinate of the peak significant activation (*p* < 0.05, FWE corrected) and cluster extent								
	CSM-still^1^				CSL-still^2^				Integration = [CSLM – (CSM + CSL)]^3^			
	Voxels	*x*	*y*	*z*	Voxels	*x*	*y*	*z*	Voxels	*x*	*y*	*z*
L Occipito-temporal junction (MT/V5) L Middle occipital gyrus L Middle temporal gyrus L Inferior occipital gyrus L Superior temporal gyrus L Inferior parietal lobe	3641	–54	–72	4								
L Occipito-temporal junction (MT/V5) L Middle occipital gyrus L Middle temporal gyrus									97	–54	–78	4
L Middle temporal gyrus	1267	–62	–26	–4								
R Occipito-temporal junction (MT/V5) R Middle temporal gyrus R Inferior temporal gyrus	1186	54	–68	–2								
L inferior frontal gyrus *pars opercularis* (BA 44) L precentral gyrus	305	–46	6	18	18	–46	6	18				
L inferior frontal gyrus *pars triangularis* (BA 45)	296	–50	32	4	81	–52	32	4				
L Precentral gyrus (BA 6)	193	–52	–2	46	10	–54	–2	48				
R Middle temporal gyrus	164	52	–36	2	285	50	–34	0				
Supplementary motor area (BA 6)	76	–4	6	64	16	–2	6	64				
R Inferior temporal gyrus	39	50	–44	–28								

*^1^Brain regions more activated during CSM perception of words compared to still control condition. ^2^Brain regions more activated during CSL perception of words compared to still control condition. ^3^Brain regions activated during integration of CSLM: [CSLM > (CSM + CSL)] Ω (CSM > still)Ω(CSL > still). L, Left; R, Right; BA, Brodmann area; *x, y, z* are standard stereotaxic coordinates in the MNI (Montreal Neurological Institute) system. Activations in CSM– still and CSL – still are reported at *p* < 0.05 whole brain corrected. Activations of CSLM integration are uncorrected for multiple comparisons; *p* < 0.001. CSLM, Cued Speech (oral + manual); CSM, Cued Speech (manual only); CSL, Cued Speech (oral only).*

#### Importance of Labial and Manual Information? Comparison of Two Conjunction Analyses Within CS: CSM – CSLM and CSL – CSLM

Finally, we wondered whether one source of information is more important in CSLM perception for deaf CS users, as for AV speech in hearing people. Indeed, the primary processing of information in speech AV decoding comes from the auditory source, whereas the contribution of visual speech to the final percept directly depends on the ambiguity of the auditory information (Summerfield, 1987). In CSLM production, it has been demonstrated that the manual cues movement anticipates the lip movements, and that this anticipation is used by the CS-perceivers to reduce the uncertainty about the phonemes pronounced (Attina et al., 2004, 2006; Troille, 2009). This suggests the possibility that manual cues are dominant in CSLM processing, while lipreading would have a (secondary) role associated to the disambiguation of the percept.

**FIGURE 7:**
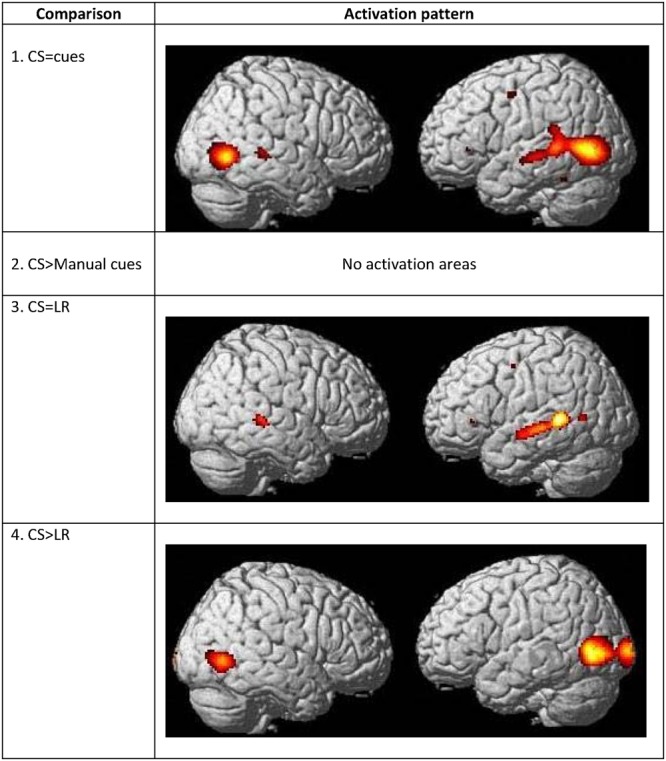
**Commonalities and differences within deaf group between lipreading and manual cues conditions.** (1) Conjunction analysis between CSLM [CSLM – still] and CSM [manual cues – still] perception of words. (2) Higher activations during CSLM perception of words [CSLM – still] compared to manual cue perception of words [CSM – still]. (3) Conjunction analysis between CSLM [CSLM – still] and CSL (lipreading) perception of words [CSL – still]. (4) Higher activations during CSLM perception of words [CSLM – still] compared to CSL (lipreading) perception of words [CSL – still]. CSLM, Cued Speech (oral + manual); CSM, Cued Speech (manual only); CSL, Cued Speech (oral only); *p* < 0.05 whole brain corrected.

A conjunction analysis between CSLM and the CSM conditions was performed and revealed widely overlapping neural activity, especially in the bilateral occipito-temporal junction, the left superior and middle temporal and inferior parietal lobes (**Figure 7** and **Table 7**).

**Table 7:** Commonalities and differences between conditions within deaf CS group^∗^, related to **Figure 7**.

Cerebral region – cluster	Coordinate of the peak significant activation (*p* < 0.05, FWE corrected) and cluster extent^∗^								
	CSLM = CSM^1^				CSLM = CSL (LR)^2^				CSLM > CSL (LR)^3^			
	Voxels	*x*	*y*	*z*	Voxels	*x*	*y*	*z*	Voxels	*x*	*y*	*z*
L Occipito-temporal junction (MT/V5) L Middle occipital gyrus L Inferior occipital gyrus L Inferior temporal gyrus L Middle temporal gyrus L Superior temporal gyrus Left Inferior parietal lobe	2131	–54	–78	9					1152	–52	–78	2
Visual primary cortex (BA 17/18)									610	–24	–102	4
R Occipito-temporal junction (MT/V5) R Inferior occipital gyrus R Inferior temporal gyrus R Middle temporal gyrus	800	54	–72	3					520	54	68	–2
L Middle temporal gyrus					671	–64	–22	–4				
R Middle temporal gyrus	117	52	–34	2	132	52	–34	2				
L Precentral gyrus (BA 6)	22	–55	–5	49	5	–54	–2	48				
L Inferior temporal gyrus	12	–50	–48	–24								
L Inferior frontal gyrus *pars triangularis*	4	–50	32	2	4	–50	32	2				

*^1^Regions commonly activated in CSLM [CSLM – still] and CSM [CSM– still] perception of words. ^2^Regions commonly activated in CSLM [CSLM – still] and CSL (lipreading) [CSL – still] perception of words. ^3^Brain regions more activated during CSLM perception of words [CSLM– still] compared to CSL perception of words [CSL– still]. L, Left; R, Right; BA, Brodmann area; *x, y, z* are standard stereotaxic coordinates in the MNI (Montreal Neurological Institute) system. All activations reported at *p* < 0.05 whole brain corrected. CSLM, Cued Speech (oral + manual); CSM, Cued Speech (manual only); CSL, Cued Speech (oral only); LR, lipreading. ^∗^There is no cluster significantly more activated when comparing CSLM to CSM in either way (i.e., CSLM – CSM or CSM-CSLM).*

**FIGURE 8:**
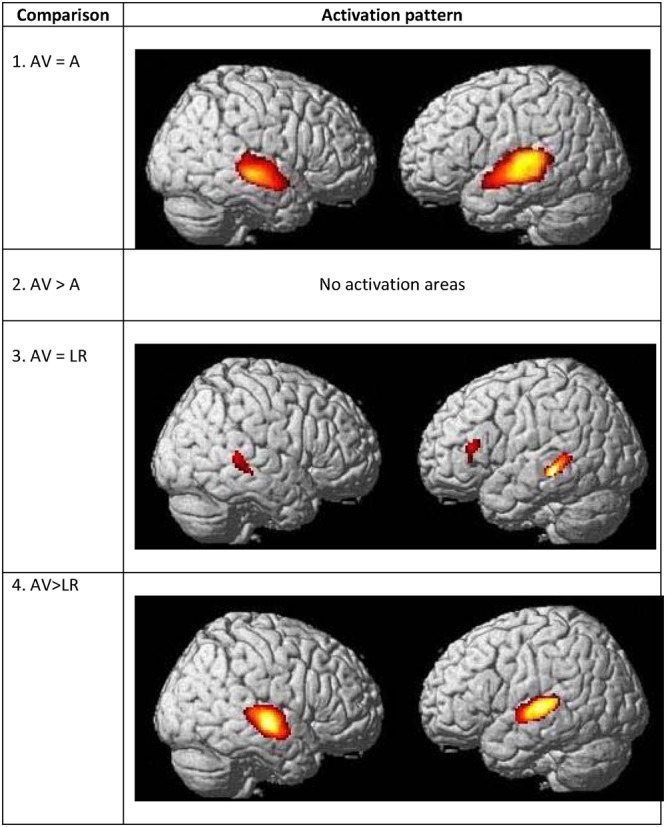
**Commonalities and differences within hearing group between lipreading and auditory conditions.** (1) Conjunction analysis between speech AV [speech AV – still] and speech auditory [speech A – still] perception of words. (2) Higher activations during speech AV perception of words [speech AV – still] compared to speech auditory [speech A – still] perception of words. (3) Conjunction analysis between speech AV [speech AV – still] and speech visual (lipreading) [speech visual – still] perception of words. (4) Higher activations during speech AV perception of words [speech AV – still] compared to speech visual (lipreading) [speech visual – still] perception of words. AV, audiovisual speech in hearing speakers; A, auditory; *p* < 0.05 whole brain corrected.

The number of overlapping voxels in this conjunction represented 94% of the voxels activated in the CSLM network. Conversely, the conjunction analysis between CSLM and CSL conditions revealed a low overlap with only 25% of the CSLM network activated (mostly in the left middle temporal gyrus). Although this observation of higher overlap with the CSLM network was made at the descriptive level only, these conclusions are reinforced by subtraction analyses that failed to reveal significant differences between CSLM and CSM conditions, whereas extended, higher activation in the CSLM than in the CSL condition was found in the bilateral occipito-temporal junction. The great overlapping activity of CSLM and CSM might be linked to the common kinematic signature created by the movements in hands. However, our results may also suggest that the processing of manual cues might play a primary (dominant) role in CSLM decoding, while lipreading may play a second role. This makes CSLM even more akin to speech AV processing : similar analyses computed in the NH group revealed a high similarity between processing of speech AV and speech auditory conditions (68% overlap), but not between speech AV and lipreading (10% overlap), and subtractions showed larger differences between speech AV and lipreading conditions (**Figure 8** and **Table 8**).

**Table 8:** Commonalities and differences between conditions within NH group^∗^, related to **Figure 8**.

Cerebral region – cluster	Coordinate of the peak significant activation (*p* < 0.05, FWE corrected) and cluster extent^∗^											
	Speech AV = speech A^1^				Speech AV = speech visual^2^				Speech AV > speech visual^3^			
	Voxels	*x*	*y*	*z*	Voxels	*x*	*y*	*z*	Voxels	*x*	*y*	*z*
L Middle temporal gyrus L Superior temporal gyrus *Including primary auditory cortex* L Inferior parietal lobe	2427	–62	–26	6					1416	–48	–26	2
L Middle temporal gyrus					315	–58	–36	–2				
R Middle temporal gyrus R Superior temporal gyrus R Inferior parietal lobe	1344	64	–14	–8					1281	63	–17	–1
R Middle temporal gyrus					176	50	–37	2				
R Superior temporal gyrus	8	48	–34	10					11	44	–28	8
L Inferior frontal gyrus *pars triangularis*					83	–50	28	14				

*^1^Regions commonly activated in speech AV [speech AV – still] and auditory [speech A– still] perception of words. ^2^Regions commonly activated in speech AV [speech AV vs. still control condition] and speech visual (lipreading) [speech visual – still] perception of words. ^3^Brain regions more activated during speech AV perception of words [speech AV– still] compared to speech visual perception of words [speech visual – still]. L, Left; R, Right; x, y, z are standard stereotaxic coordinates in the MNI (Montreal Neurological Institute) system. All activations reported at p < 0.05 whole brain corrected. P < 0.05 whole brain corrected. AV, audio-visual; A, auditory; ^∗^There is no cluster significantly more activated when comparing speech AV to speech A (auditory alone) in either way (i.e., speech AV-speech A or speech A-speech AV).*

## Discussion

The current study compared the fMRI activations of CS processing by deaf participants with that of speech AV processing by hearing participants, with identical spoken words, presented either in CS (i.e., lipreading and manual cues) for deaf CS participants (CS group) or audio-visually for normally hearing participants (NH group). Both groups of participants had French as their native language. Our study is the first neuroimaging investigation of deaf people who had full sensory access to the phonemic and syllabic content of spoken speech, through the visual modality.

Our first research question concerned the similarities and differences between the processing of CSLM by early CS-users and the processing of speech AV speech by hearing participants. Our results show that the perception of oral language delivered through CSLM activates secondary auditory cortices in the MTG and STG together with IPL. This activation represents most of the overlapping activation regions found during CSLM and AV speech processing (see **Figure 3** and **Table 3**). In AV speech processing, MTG and STG are parts of a structure in the auditory ventral stream (Hickok and Poeppel, 2004) that serves as an interface between sound based representations of speech (Binder et al., 2000), visual speech (see Bernstein and Liebenthal, 2014 for a review) and widely distributed conceptual representations (Damasio, 1992). However, in CSLM, activity in MTG and STG cannot be linked to any kind of acoustic processing. Since earlier studies showed a very accurate CS perception and comprehension in deaf early CS users (Leybaert and Charlier, 1996; Alegria et al., 1999; Leybaert and D’Hondt, 2003; Aparicio et al., 2012; Colin et al., 2013), activation of the secondary auditory cortex might be linked to an interface between phonetic visual speech codes conveyed by lip movements/manual cues and conceptual representations.

Differences in brain activation between CSLM and AV speech showed that the neural basis of speech perception in CSLM is shifted toward posterior regions of the brain (parieto-occipital regions) as compared to AV. Some studies have reported more posterior language processing in less experienced language users, deaf (Mayberry et al., 2011) and not deaf (Brown et al., 2005; Gaffrey et al., 2007). However, our CS participants were exposed to visual representation of spoken language as their native language. Our deaf participants had all a good level of comprehension of CS (see participants). This suggests that activations presented here are not the consequence of poor language processing.

Interestingly, the common component of the speech AV and CSLM brain networks is located in the posterior portion of the STG. In contrast, the anterior part of STG (including the auditory primary cortex) is *only* activated in AV speech processing (see **Figure 3** and **Table 3**). We surmise here a functional subdivision of the superior temporal lobe for speech processing, with the anterior part supporting speech processing based on sounds (i.e., phonetic codes), and the posterior portion involved in speech processing integrating information provided by different modalities (visual, auditory, and even somatosensory). Indeed, previous studies have shown that the multisensory posterior STG can be involved either in acoustic-phonetic processing (Hickok and Poeppel, 2007), in phonological mediation for lipreading (Paulesu et al., 2003) or in simultaneous auditory-tactile stimulation (Beauchamp et al., 2008). Moreover, activation of STG also appeared during SL processing in deaf people suggesting that neural activity in posterior STG is linked to linguistic processing beyond auditory representation (Petitto et al., 2000; MacSweeney et al., 2008; Xu et al., 2009). The present findings also indicate that the multisensory posterior STG could be activated by the processing of language cues *within a single modality* (here: visual), at least when these visual cues provide meaning for the individual as it was the case with the words presented in this experiment. Given previous findings showing that the posterior STG is activated when participants observe symbolic gestures (Xu et al., 2009), our data support the hypothesis that this region responds to meaningful manual-lipread stimuli in deaf CS-users as well as vocal-auditory stimuli in hearing AV participants. Our results thus provide converging evidence with these previous neuroimaging studies, but also add the new information about involvement of posterior STG in the processing of CSLM. Finally, there is a greater activation of occipital gyrus in CSLM compared to AV. This might reflect the greater kinematic information supplied by the hands and lip movement in CS.

It should be noted that activation in posterior STG during CSLM processing could also be explained by the fact that our CS participants were all deaf. These two interpretations are not easy to disentangle. Olulade et al. (2014) showed that cortical plasticity resulting from deafness depends on language experience for auditory and visual areas. If normally hearing participants who are CS-users (parents, teachers, educators of deaf CS-users) also showed activation of posterior STS, the hypothesis that multi-signal integration inside the visual modality occurs in this region of the brain would be reinforced.

Our second research question concerned the neural basis of integration of manual cues and lipread information. In CSLM, the integration is mandatory and concerns two types of dynamic information in the visual modality (i.e., visible movement of the labial and manual articulators). In contrast, in AV speech, integration concerns information in two different modalities: auditory and visual. Our integration analysis suggests that left MT/V5 plays a role in the integration of lipread and manual CS information. Note that this region has been already linked to speech processing (Jones and Callan, 2003; Sekiyama et al., 2003) and to processing of visual movements in SL (Capek et al., 2008). In addition, it has been shown that use of a visuospatial language like SL impacts the recruitment of the MT/V5 during motion visual processing. Deaf and hearing signers show a greater activation of the left MT/V5 than the right, while hearing non-signers show the opposite pattern (Bavelier et al., 2001; Bavelier and Neville, 2002), indicating that lateralization of MT/V5 is sensitive to language experience. Given that speech input in CS is primarily perceived through visual occipital networks, one may surmise that integration in the MT/V5 area is an early step in CSLM processing. This conception seems logical considering that manual cues and lips cues need to be processed before unambiguous decoding of speech (Alegria and Lechat, 2005). Importantly, our integration analysis, unlike the other analyses (e.g., subtractions) conducted in this study, controls for perception of movements related to CS production, since the comparison is made between CSLM and the sum of manual cues and lipreading. However, we cannot firmly conclude that integration occurring in MT/V5 is *linguistic* because some (or even all) of this integration might be non-linguistic, or even attentional (O’Craven et al., 1997). To support the hypothesis of a linguistic integration in MT/V5, future studies should compare these activation patterns with those coming from a control group that does not use or understand CS, presented with the same set of stimuli. In relation to the role of MT/V5 during CSLM perception, our PPI analyses yielded increased connectivity between left MT/V5 and speech processing regions located in the left temporal lobe. This suggests a cooperative activity between these areas in the treatment of CS components.

In addition, PPI analysis showed a functional inter-hemispheric differentiation in CS users: left MT/V5 interacts with left temporal lobes linked to speech processing whereas right MT/V5 interacts mostly with occipital areas, including left MT/V5, linked to visual perception and motion processing.

Our third research question concerned the relative activation created by CSM and by CSL compared to the activation created by CSLM. If the manual cues play a leading role in the processing of CSLM, one could expect a greater overlap between the activation created by the CSM and CS conditions, compared to a lower overlap between the activation created by CSL and CSLM. The conjunction analyses between CSLM and CSM conditions, on the one hand, and CSLM and CSL conditions on the other hand may be associated to a dominant role for the manual cues. Indeed, the almost complete overlap between CSLM and CSM networks is compatible with the view that the manual cues first provide phonological information, and lipreading intervenes subsequently in order to further disambiguate the linguistic message. This conclusion is in good agreement with three types of behavioral data. First, temporal analyses of CS production have shown that manual cues are produced temporally in advance to the lips (Attina et al., 2004, 2006; Troille, 2009). Second, when lipreading and manual cues are incongruent (e.g., pronouncing with the lips the phoneme /v/ with handshape 1 coding /d/, /p/ /j/ phonemes), most of the answers from the perceiver are related to the manual cues and not to lipreading, especially when the participant is an early CS user (Alegria and Lechat, 2005; Bayard et al., 2014, 2015). Third, deaf people who are early CS-users often succeed in daily natural communication with other CS users by producing manual cues alone, without lipreading. In this case, the full meaning of the message would be completed by the context (Weill, 2011). While the set of manual cues was initially originally created with the aim of disambiguating lipreading (Cornett, 1967), the present data support the hypothesis that *it is lipreading which disambiguates manual cues*, thus presenting a *topsy-turvy* vision of CS (Attina et al., 2004). One possible reason for this phenomenon is that in speech processing the perceiver takes a “preference” for decoding from those elements that are perceptually most distinguishable (i.e., the manual cues in CS for deaf participants or audio in AV for hearing participants). In case of deaf CS perceivers, it must also kept in mind that manual CS cues are executed and visually available to the perceiver a very short time before lip movements (Attina et al., 2004). At this step one could not also exclude that prior hand cues may reduce ambiguity before mouth shapes processing.

Interestingly, manual cues were associated with increased activity in left superior and middle temporal gyrus (**Figure 6** and **Table 6**), a brain zone typically activated during the processing of natural languages like spoken languages and signed languages. However, manual cues are completely artificial gestures, not resulting from any evolutionary process. This suggests that invented manual gestures that convey linguistic information may become processed in the same areas as other articulatory gestures previously integrated in the human language through the natural evolutionary process of communication.

These findings should be investigated in further studies to better understand the degree of dependency of language processing on speech features. For example, in order to better determine the brain areas subtending speech processing, one could conduct a similar study of speech perception, which would use a control condition containing pseudospeech movements through manual cues and lips, instead of a still control condition. The pseudospeech would be phonologically plausible but meaningless. This condition would enable us to dissociate brain areas linked to phonological processing from those linked to lexical processing in visual CS. Another interesting study would be to investigate the neural correlates of incongruent lip and manual cue movements (for example, a mouthed syllable /va/ accompanied by handshape 1 [/p, d, j/]), i.e., a McGurk-like effect experiment (Alegria and Lechat, 2005; Bayard et al., 2014, 2015). In CS, this would increase our understanding of the integration of visual speech features in deaf participants. Finally, we are interested in examining how manual cues are integrated with AV speech in deaf people fitted with a cochlear implant (Bayard et al., 2014, 2015). As mentioned, the articulatory movement of the hand precedes the mouth opening and the emission of sound and may therefore predict aspects of the lipread and the auditory signals, especially when the AV signal is ambiguous (e.g., visual /k/). The amount and nature of visual information extracted from the hand may initiate the speech-processing system, in which an abstract representation is activated through visual inputs, up to the point of explicitly registering auditory input. If this speculation appears to be correct, it means that processing of CS gestures could help deaf children fitted with a cochlear implant to discriminate, identify or interpret the new arriving sounds. Integration across modalities would allow the STG to help individuals with cochlear implants in discrimination, identification or interpreting the ambiguous auditory cues delivered by the cochlear implant.

At a more general level, our data about the neural processing of CS increase our general knowledge on how do deaf native users of CS process visual speech information. Compared to normally hearing individuals, deaf native users of spoken language have fewer areas of anatomical differences than do deaf native users of ASL (Olulade et al., 2014). The neural processes involved in CS and in AV speech seem, to a certain extent, also similar.

## Conclusion

In this article, we report results from the first neuroimaging study of CS processing, a mode of communication in which the syllables and phonemes of a spoken language are conveyed solely through the visual modality in the absence of either speech or hearing. First, we found that activation patterns in the secondary auditory cortex (i.e., temporal lobes) in visual CS perception and speech AV perception confirm the existence of a common language brain system for spoken languages, regardless of the sensory input modality. Second, our PPI and integration analyses suggest that MT/V5, a region classically associated with visual motion processing, exerts an active influence on the integration of hands and mouth. However, based on our analysis, we cannot conclude that this integration is merely linguistic. Finally, findings from our study suggest that the manual cues may dominate in the speech perception of skilled CS.

## Author Contributions

MA, PP, and JL worked in the conception of the research and the redaction of the manuscript. BC has collaborated in the creation of the stimulus material. DB and MK have collaborated for the MRI data acquisition

## Conflict of Interest Statement

The authors declare that the research was conducted in the absence of any commercial or financial relationships that could be construed as a potential conflict of interest.
