# Supplementary material for: The Neural Basis of Speech Perception through Lipreading and Manual Cues: Evidence from Deaf Native Users of Cued Speech
Source: Front Psychol. 2017 Mar 30;8:426. doi: 10.3389/fpsyg.2017.00426 (PMC5371603; doi:10.3389/fpsyg.2017.00426)
Supplement: Supplementary file 1 [file Data_Sheet_1.docx]

ANNEX

**Words used in this study**

appel [apɛl]

armée [aʁme]

avion [avjɔ̃]

bateau [bato]

bureau [byʁo]

café [kafe]

camion [kamjɔ̃]

cerveau [sɛʁvo]

chapeau [ʃapo]

couloir[kulwaʁ]

debout [dəbu]

demain [dəmɛ̃]

demi [dəmi]

depuis [dəpɥi]

espoir [ɛspwaʁ]

fatigue [fatiɡ]

fermer [fɛʁme]

finir [finiʁ]

forêt [fɔʁɛ]

fumée [fyme]

hiver [ivɛʁ]

machine [maʃin]

manteau [mɑ̃to]

marché [maʁʃe]

mentir [mɑ̃tiʁ]

métier [metje]

million [miljɔ̃]

monsieur [məsjø]

monter [mɔ̃te]

morceau [mɔʁso]

mourir [muʁiʁ]

niveau [nivo]

papa [papa]

papier [papje]

pardon [paʁdɔ̃]

parfois [paʁfwa]

passion [pasjɔ̃]

payer [peje]

pays [pei]

poisson [pwasɔ̃]

photo [fɔto]

projet [pʁɔʒɛ]

repos [ʁəpo]

sommeil [sɔmɛj]

tableau [tablo]
